# miR-103/107 prolong Wnt/β-catenin signaling and colorectal cancer stemness by targeting Axin2

**DOI:** 10.1038/s41598-019-41053-z

**Published:** 2019-07-04

**Authors:** Hsin-Yi Chen, Yaw-Dong Lang, Han-Nan Lin, Yun-Ru Liu, Chun-Chieh Liao, André Wendindondé Nana, Yun Yen, Ruey-Hwa Chen

**Affiliations:** 1 Ph.D Program for Cancer Molecular Biology and Drug Discovery, College of Medical Science and Technology, Taipei Medical University, Taipei, Taiwan; 2 Institute of Biological Chemistry, Academia Sinica, Taipei, Taiwan; 3 Institute of Molecular Medicine, College of Medicine, National Taiwan University, Taipei, Taiwan; 4 Institute of Biomedical Science, Academia Sinica, Taipei, Taiwan; 5 Institute of Biochemical Sciences, College of Life Science, National Taiwan University, Taipei, Taiwan; 6 Office of Human Research, Taipei Medical University, Taipei, Taiwan; 7 Graduate Institute of Cancer Biology and Drug Discovery, College of Medical Science and Technology, Taipei Medical University, Taipei, Taiwan; 8 TMU Research Center of Cancer Translational Medicine, Taipei, Taiwan

**Keywords:** Colon cancer, Metastasis

## Abstract

Cancer stemness drives tumor initiation, progression, metastasis, recurrence, and therapy resistance. However, mechanisms that potentiate the acquisition and maintenance of stemness fate of cancer cells remain incompletely understood. Here, we show that miR-103/107 stimulate multiple stem-like features in colorectal cancer, including expression of stem-like markers, appearance of side-population cells, and capabilities in self-renewal, tumor initiation, recurrence, and chemoresistance. Mechanistically, these stemness-promoting functions are mediated by miR-103/107-dependent repression of Axin2, a negative feedback regulator of Wnt/β-catenin signaling. Through inhibiting Axin2, miR-103/107 trigger a prolonged duration of Wnt/β-catenin signaling and a sustained induction of Wnt responsive genes. In colorectal cancer patients, miR-103/107 expression correlates inversely with Axin2 expression and a signature of miR-103/107 high and Axin2 low expression profile correlates with poor prognosis. Together, our study identifies a novel function of miR-103/107 in promoting colorectal cancer stemness by targeting Axin2 and elucidates the clinical relevance and prognostic value of this axis in colorectal cancer.

## Introduction

Colorectal cancer (CRC) is the third most common cancer type in the world^1^. Compelling evidence has established that cancer stem cells (CSCs) are a subset of tumor cells with self-renewal capability and multilineage-differentiation potential^2,3^. More importantly, CSCs show more potent abilities in tumor initiation, metastatic progression, recurrence, and therapy resistance than bulk cancer cells, and thus become a major hurdle for cancer eradication^4,5^. Unraveling the molecular mechanisms and signaling pathways driving cancer stemness would be beneficial for identifying invaluable diagnostic, prognostic, and therapeutic targets for clinical applications. However, the factors and mechanisms that determine the acquisition and maintenance of stem-like characters of tumor cells remain incompletely understood.

The Wnt/β-catenin pathway is crucial for the maintenance of stem cells within normal intestine and CSC population in CRC^6,7^. The key step of Wnt/β-catenin pathway involves β-catenin stabilization. In the absence of stimulation, β-catenin is phosphorylated by the destruction complex containing adenomatous polyposis coli (APC), casein kinase 1, glycogen synthase kinase 3β, and Axin1/2, and subsequently undergoes ubiquitin-dependent proteolysis^8^. Binding of Wnt ligands to their cognate receptors triggers the recruitment of Axin1 to the receptor-associated scaffold protein Dvl, which leads to the disassembly of destruction complex and the accumulation and nuclear translocation of β-catenin^9^. In the nucleus, β-catenin associates with the lymphoid enhancer factor/T-cell factor (LEF/TCF) family of transcription factors, converting them from transcription repressors to activators, thereby activating a wide range of Wnt responsive genes^10–12^.

The Wnt responsive genes include Wnt pathway effectors and negative feedback regulators^13^. Axin2 is a Wnt-induced negative feedback regulator to control timely termination of Wnt-induced transcriptional responses. Compared to the constitutively expressed Axin1, Axin2 interacts less efficiently with Dvl2 and therefore can facilitate β-catenin degradation even in the presence of upstream signaling, a feature important for negative feedback regulation. In line with this notion, knockdown of Axin2, but not Axin1, increases Wnt3a-induced β-catenin dephosphorylation, nuclear translocation, and TCF/β-catenin reporter activity^14^. Conversely, pharmacological stabilization of Axin2 blocks aberrant β-catenin activity caused by reduction in APC function^15^. Consistent with its function in suppressing Wnt signaling, Axin2 downregulation and truncation have been frequently found in CRC and sessile serrated adenomas, a colon cancer precursor lesion distinct from the typical adenomas^16,17^. Although hypermethylation of *Axin2* promoter is observed in CRC, Axin2 downregulation in the absence of promoter hypermethylation has also been reported^16,17^, suggesting the existence of an additional layer of Axin2 downregulation mechanism in CRC.

miR-103 and miR-107, belonging to the miR-103/107 family by sharing with the same seed sequences, function as “oncomirs” in many types of cancers. Clinically, miR-103/107 overexpression in tumor tissues or circulation is associated with poor survival, recurrence, and lymph node metastasis in various cancers, including colorectal, breast, esophageal, and gastric cancers^18–22^. Mechanistically, miR-103/107 enhance CRC growth and metastasis via downregulating LATS2, DAPK, and KLF4^23,24^. In breast cancer, miR-103/107 stimulate metastasis and angiogenesis by targeting Dicer and Ago1, respectively^25,26^. Additionally, miR-107 promotes breast tumor progression by targeting let-7^27^. Despite these tumor-promoting functions, the impact of miR-103/107 on cancer stemness is still unknown. Here, we report that miR-103/107 target Axin2 to prolong the duration of Wnt/β-catenin signaling, thereby conferring stem-like phenotypes of CRC cells. This miR-103/107-dependent Axin2 downregulation potentiates multiple stem-like features of CRC cells. Furthermore, a signature of miR-103/107 high and Axin2 low expression profile correlates with recurrence and poor overall survival in patients with CRC. Our study reveals a pivotal role of miR-103/107-dependent Axin2 repression in CRC stemness.

## Results

### miR-103/107 promote CRC stem-like properties

**Figure 1:**
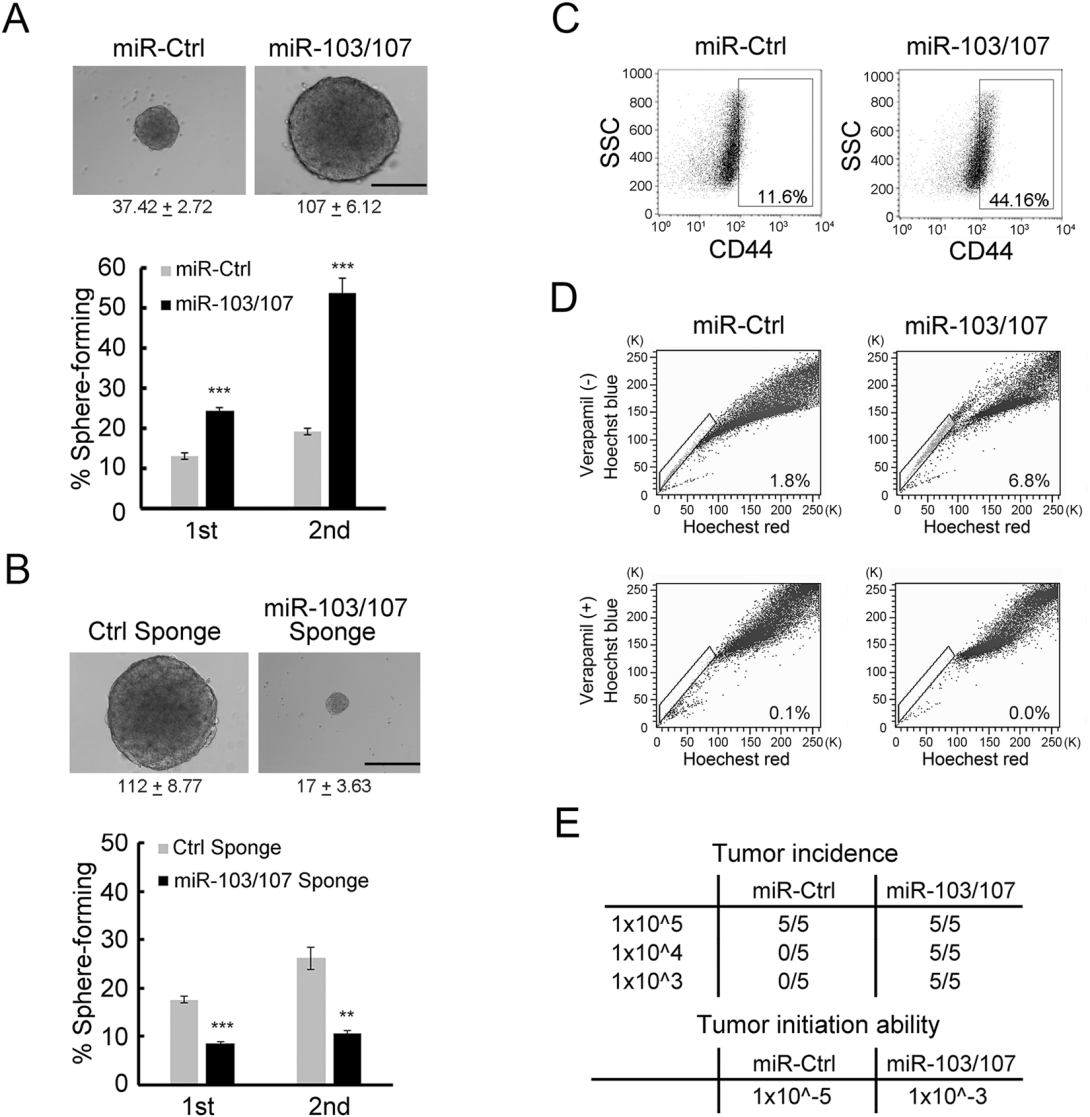
miR-103/107 promote stem-like properties of CRC cells. (**A**,**B**) Tumor sphere-forming abilities of HCT116 cells stably expressing indicated miRNAs (**A**) or miRNA sponges (**B**). Representative images of tumor spheres and sphere sizes (calculated from 30 cells per group per experiment) from indicated cells are shown on the upper panels. The images were taken at 2 (**A**) and 3 (**B**) weeks after cell plating. Quantitative data of sphere numbers are shown on the bottom panels. Scale bars, 50 mm. Data are mean ± S.D. **P < 0.005, ***P < 0.0005, n = 3. (**C**,**D**) HCT116 cells expressing indicated miRNAs were assayed by flow cytometry for cell surface expression of stem-like marker CD44 (**C**) and side-population cells (**D**). (**E**) Tumor-initiation ability was measured by subcutaneously transplanting HCT116 derivatives into nude mice at a density of 10^5^, 10^4^, or 10^3^. Seven weeks after transplantation, tumor incidence and tumor-initiation ability (incidence of tumor formation per cell number) were monitored.

Our previous study identified an association of miR-103/107 expression with poor prognosis of CRC patients^23^. Since cancer stemness has been tightly linked to poor prognosis by promoting recurrence, metastasis, and treatment resistance, we investigated the impact of miR-103/107 on several stemness-related characteristics of CRC cells, including self-renewal ability, expression of CRC stem-like marker, appearance of side-population cells and tumor-initiation potential. Using a previously established CRC cell system which stably expresses miR-103/107 or a control miRNA^23^, we found that miR-103/107 overexpression enhanced the formation of floating tumor spheroids both in number and size under anchorage-independent and serum-free conditions (Fig. 1A). To test the propagation potential of these spheres, we dissociated the primary spheres for testing the formation of secondary spheres. Again, miR-103/107 overexpression led to a significant increase in the formation of secondary spheres. In the reciprocal experiment, we depleted miR-103/107 in HCT116 cells using sponge technology^28^. Expression of miR-103/107 sponge successfully de-repressed DAPK and KLF4, two established targets of miR-103/107 (Fig. S1), indicating the effectiveness of this approach. Importantly, we observed an inhibitory effect of miR-103/107 sponge on the formation of both primary and secondary spheres (Fig. 1B). Consistent with these findings, miR-103/107 overexpression in CRC cells increased the expression of stem-like marker CD44 and the appearance of side-population cells, a stem-like feature (Fig. 1C,D). Furthermore, miR-103/107 overexpression led to a marked enhancement in the tumor formation capability in nude mice receiving a low number of tumor cells (Fig. 1E). The tumor-initiation capability (incidence of tumor formation per cell number) of miR-103/107 expressing HCT116 cells was 100-fold higher than that of control cells (Fig. 1E). Together, these findings identify a role of miR-103/107 in promoting stem-like properties of CRC cells.

### miR-103/107 promote CRC stemness via Wnt/β-catenin signaling

**Figure 2:**
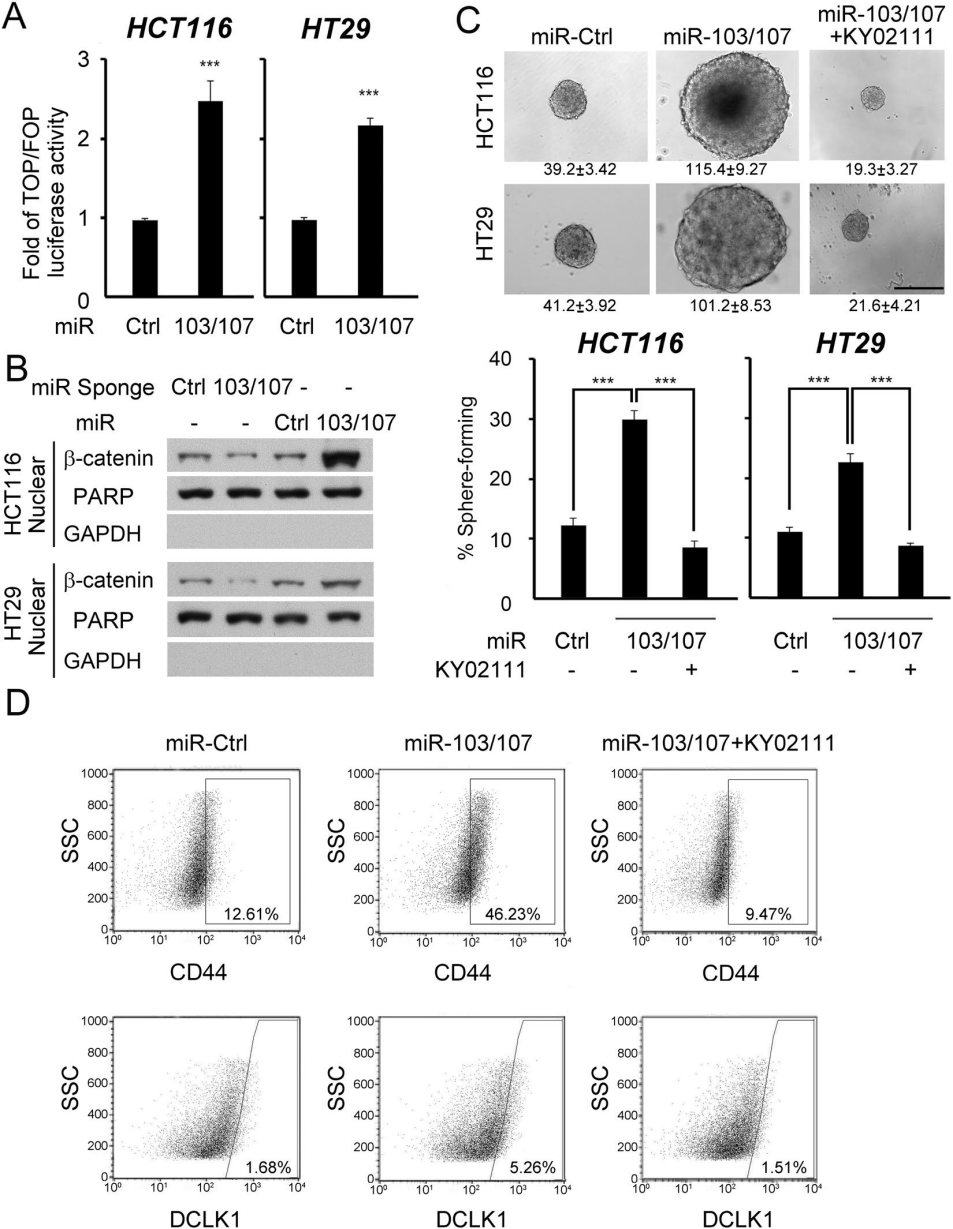
MiR-103/107 promote stem-like properties via Wnt signaling. (**A**) TCF/β-catenin reporter activity in HCT116 or HT29 cells stably expressing indicated miRNAs was assayed by TOP/FOP flash luciferase analysis. HT29 stable lines were established in our previous study^23^. (**B**) Nuclear β-catenin abundance in HCT116 (upper panel) or HT29 (bottom panel) cells stably expressing indicated miRNAs or miRNA sponges was measured by immunoblot analysis. PARP1 was used as a nuclear marker and GAPDH was used as a cytoplasmic marker. (**C**) Tumor sphere-forming abilities of HCT116 cells (upper panel) or HT29 (bottom panel) stably expressing indicated miRNAs in the presence or absence of KY02111 treatment. Representative images of spheres and sphere sizes (calculated from 30 spheres per group per experiment) are shown on the top panel. The images were taken at 2 weeks after cell plating. Quantitative data of sphere numbers are shown on the bottom panel. Scale bar, 50 mm. (**D**) Cell surface expression of CD44 (upper panel) or DCLK1 (bottom panel) in HCT116 cells stably expressing indicated miRNAs in the presence or absence of KY02111 treatment. All numerical data are mean ± S.D. **P < 0.005, ***P < 0.0005, n = 3.

Next, we elucidated the underlying mechanism of miR-103/107 in stimulating CRC stem-like features. Wnt/β-catenin signaling represents a major pathway in determining stem cell fate of intestine^29^ and promotes CRC stemness^30^. We thus examined the influence of miR-103/107 on Wnt/β-catenin signaling activity. As shown in Fig. 2A,B, miR-103/107 overexpression elevated β-catenin/TCF-dependent reporter activity and nuclear β-catenin abundance, whereas depletion of miR-103/107 by sponge decreased nuclear β-catenin level, both in HCT116 and HT29 cell lines. Furthermore, inhibition of Wnt signaling by a pharmacological inhibitor KY02111^31^ blocked miR-103/107-induced stem-like characters, including self-renewal potential and the expression of two stem-like markers, CD44 and DCLK1 (Fig. 2C,D). These data support a role of Wnt/β-catenin signaling in miR-103/107-enhanced CRC stem-like properties.

### Axin2 is a direct target of miR-103/107

**Figure 3:**
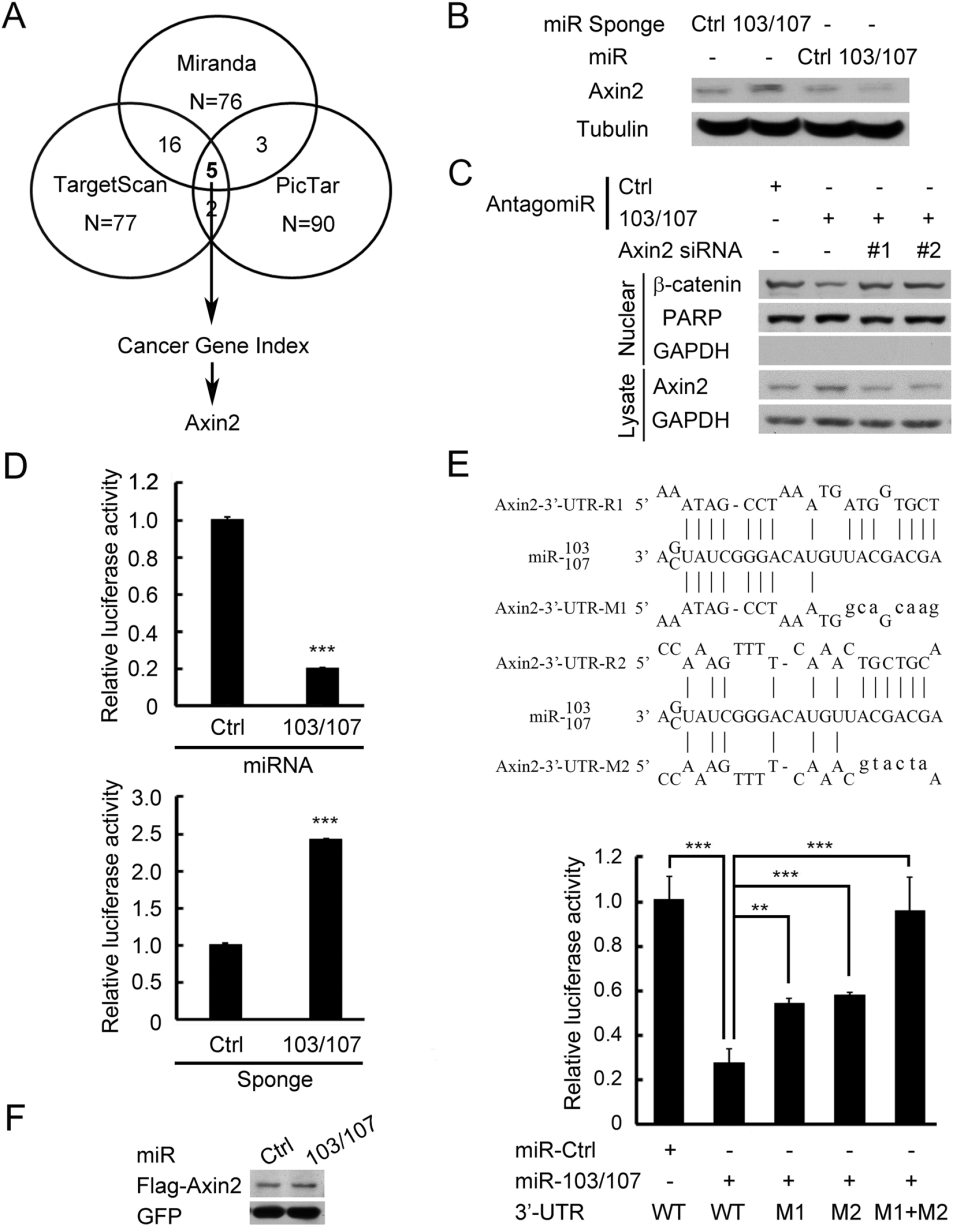
Axin2 is a direct target of miR-103/107. (**A**) Prediction of miR-103/107 targets that regulate Wnt signaling by indicated bioinformatics tools. (**B**) Immunoblot analysis of Axin2 level in HCT116 cells stably expressing indicated miRNAs or miRNA sponges. (**C**) Immunoblot analysis of Axin2 and β-catenin levels in HCT116 cells transiently transfected with indicated antagomiRs and/or siRNA. (**D**) Analysis of luciferase activity in HCT116 cells expressing *Axin2* 3′-UTR reporter and indicated miRNAs (top) or miRNA sponges (bottom). (**E**) Predicted miR-103/107-binding sequences within the *Axin2* 3′-UTR and the sequences of *Axin2* 3′-UTR mutants (Mut) used in this study (top). Luciferase activity in HCT116 cells co-transfected with indicated 3′-UTR-containing reporter constructs and miRNAs (bottom). (**F**) Immunoblot analysis of HCT116 cells transfected with indicated miRNAs and Axin2 construct without its 3′-UTR. All numerical data are mean ± S.D. **P < 0.005, ***P < 0.0005, n = 3.

Next, we used three bioinformatics algorithms, miRanda (http://www.miranda-im.org)^32^, TargetScan (http://www.targetscan.org)^33^ and PicTar (http://pictar.mdc-berlin.de)^34^, to identify potential miR-103/107 targets. Top hits predicted by all three algorithms were analyzed with Cancer Gene Index (https://wiki.nci.nih.gov/display/ICR/Cancer+Gene+Index+End+User+Documentation) to search for negative regulator of Wnt signaling. This analysis recovered Axin2 as the only candidate (Fig. 3A). We therefore investigated whether Axin2 protein level could be regulated by miR-103/107. Ectopic expression of miR-103/107 in HCT116 cells reduced the expression of Axin2, whereas blockage of endogenous miR-103/107 by sponge elevated Axin2 (Fig. 3B). Likewise, an increase in Axin2 expression was observed in HCT116 cells expressing antagomiR-103/107 (Fig. 3C). Importantly, expression of antagomiR-103/107 in HCT116 cells (Fig. 3C) and HT29 cells (Fig. S2) reduced the nuclear levels of β-catenin, and this effect was reversed by two Axin2 siRNAs. These findings support a role of endogenous miR-103/107 in β-catenin stabilization via Axin2. To substantiate that Axin2 is a direct target of miR-103/107, we generated a reporter construct in which the full-length Axin2 3′-UTR was cloned downstream of the luciferase open reading frame. Overexpression of miR-103/107 reduced the activity of this reporter, whereas antagomiR-103/107 elicited an opposite effect (Fig. 3D). The 3′-UTR of Axin2 contains two putative miR-103/107-targeting sites (Fig. 3E, upper panel). Mutagenesis of the seed sequence of either site partially inhibited the responsiveness to miR-103/107, whereas mutation of both sites fully blocked miR-103/107-induced downregulation of Axin2 reporter (Fig. 3E, lower panel). This finding indicates that the two target sites function cooperatively to mediate miR-103/107-induced Axin2 downregulation. In line with the results of reporter assays, the expressions of Flag-Axin2 from a construct lacking the 3′-UTR sequence was not affected by miR-103/107 (Fig. 3F). Thus, our data demonstrated that miR-103/107 suppress the expression of Axin2 by targeting its 3′-UTR.

### miR-103/107 prolong Wnt signaling duration by targeting Axin2

**Figure 4:**
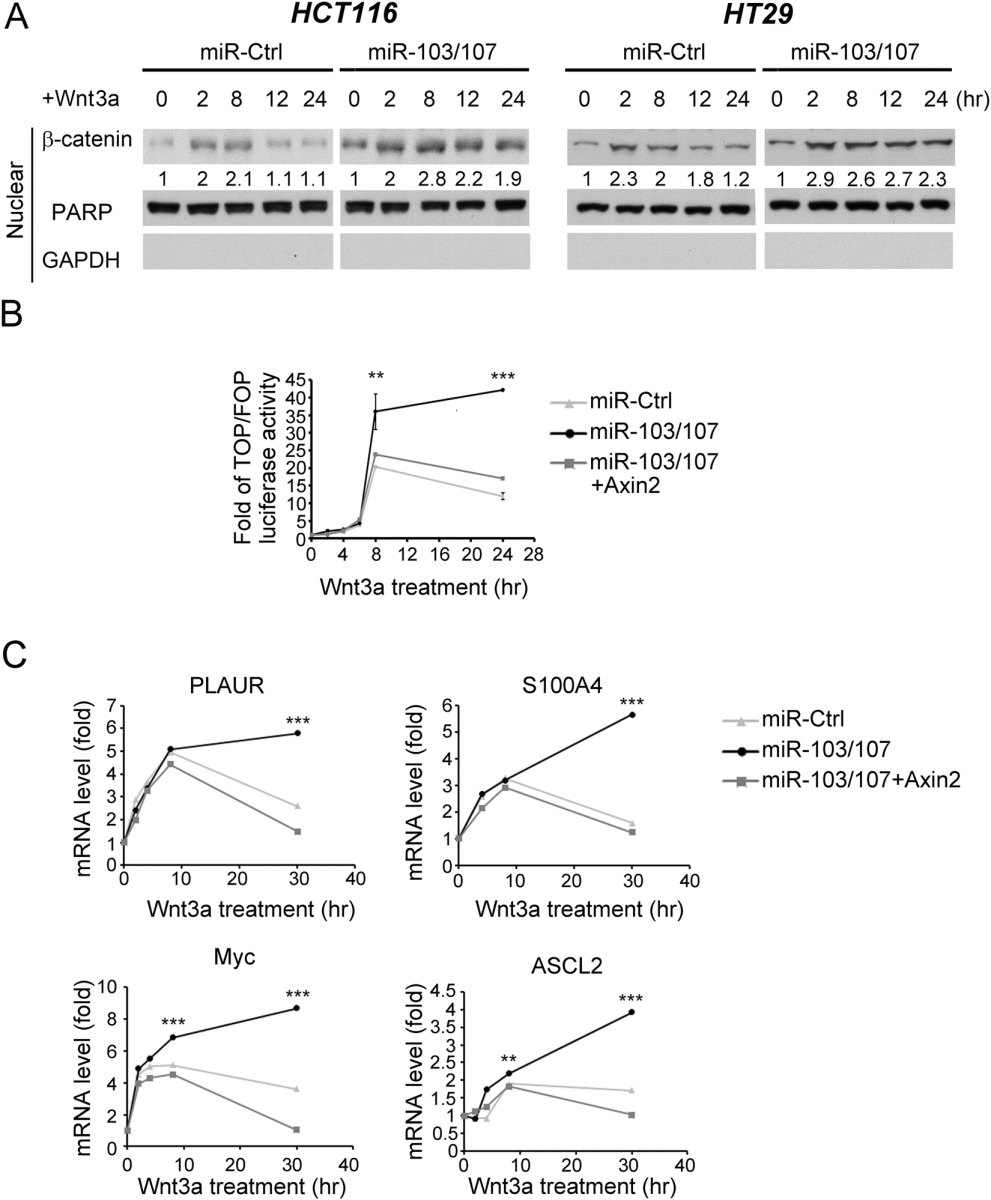
miR-103/107-Axin2 axis prolongs the duration of Wnt signaling. (**A**) Immunoblot analysis of nuclear β-catenin levels in HCT116 (left panel) or HT29 (right panel) cells expressing indicated miRNAs and treated with Wnt3a for indicated time points. HT29 stable lines were established in our previous study^23^. (**B**) Wnt reporter activity of HCT116 cells stably expressing indicated miRNAs at different time points after Wnt3a treatment was assayed by TOP/FOP flash luciferase analysis. (**C**). mRNA expression levels of indicated Wnt responsive genes in HCT116 derivatives at different time points after Wnt3a treatment was monitored by RT-qPCR. All numerical data are mean ± S.D. **P < 0.005, ***P < 0.0005, n = 3.

Axin2 is a Wnt-induced feedback regulator to enable timely termination of Wnt signaling^10,11,35^. We therefore investigated whether miR-103/107-dependent Axin2 downregulation could disrupt this negative feedback loop, thereby extending the duration of Wnt/β-catenin signaling. In HCT116 cells, Wnt3a-induced nuclear β-catenin accumulation was peaked at 8 hr, followed by a rapid decline (Fig. 4A, left panel). In miR-103/107-overexpressing cells, however, a more persistent induction of nuclear β-catenin was observed. This miR-103/107-induced extension of β-catenin induction period was recapitulated in HT29 cells (Fig. 4A, right panel). Accordingly, a more sustained induction of β-catenin/TCF reporter activity and Wnt responsive genes, including ASCL2, Myc, PLAUR and S100A4, was found in miR-103/107-overexpressing cells than control cells after Wnt3a stimulation (Fig. 4B,C). Furthermore, reconstitution of Axin2 expression in miR103/107-overexpressing cells restored the transient induction of β-catenin/TCF reporter activity and Wnt responsive genes. Thus, miR103/107 suppress the negative feedback regulation of Wnt signaling by targeting Axin2.

### Suppression of Axin2 by miR-103/107 promotes CRC stemness

**Figure 5:**
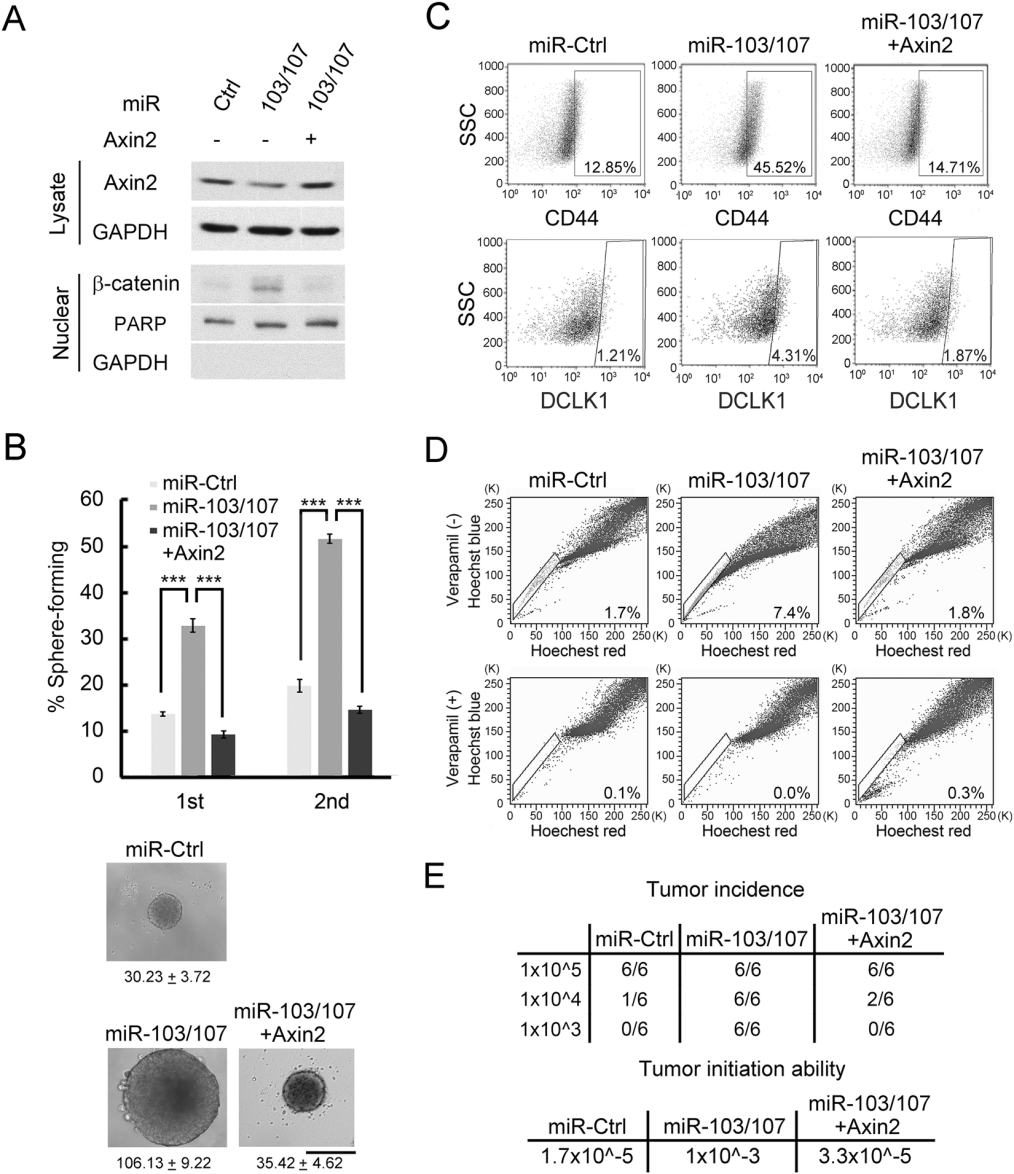
Suppression of Axin2 by miR-103/107 promotes CRC stemness. (**A**) Axin2 and nuclear β-catenin levels in HCT116 cells expressing indicated miRNAs and Axin2 construct were assayed by immunoblot analysis. (**B**) Tumor sphere-forming abilities of HCT116 derivatives as shown in (**A**). Representative of spheres and sphere sizes (calculated from 30 spheres per group per experiment) are shown on the right panel. The images were taken at 2 weeks after cell plating. Quantitative data of sphere numbers are on the left panel. Scale bar, 50 mm. Data are mean ± S.D. ***P < 0.0005, n = 3. (**C**,**D**) HCT116 derivative as shown in (**A**) were assayed by flow cytometry for cell surface expression of stem-like markers CD44 (upper panel) and DCLK1 (lower panel) (**C**) and side-population cells (**D**). (**E**) Tumor-initiation ability was measured by subcutaneously implanting HCT116 derivatives as shown in (**A**) at indicated cell numbers into nude mice. Seven weeks after transplantation, tumor incidence and tumor-initiating ability were measured.

Next, we investigated whether Axin2 downregulation contributes to miR-103/107-induced CRC stemness. To this end, HCT116 and HT29 cells stably expressing miR-103/107 and/or Axin2 were established (Figs 5A and S3A). Reexpression of Axin2 in miR-103/107-overexpressing cells blocked the ability of miR-103/107 to increase nuclear β-catenin (Figs 5A and S3A) and to stimulate the formation of tumor spheres (Figs 5B and S3B). Likewise, the effects of miR-103/107 on promoting the expression of stem-like markers CD44 and DCLK1 and the appearance of side-population cells were all abolished by Axin2 overexpression (Figs 5C,D and S3C,D). Furthermore, miR-103/107-induced tumor-initiation capability was also suppressed by Axin2 reexpression (Figs 5E and S3E). These results demonstrated a major role of Axin2 repression in miR-103/107-induced CRC stemness.

### miR-103/107-Axin2 axis contributes to chemoresistance and tumor recurrence

**Figure 6:**
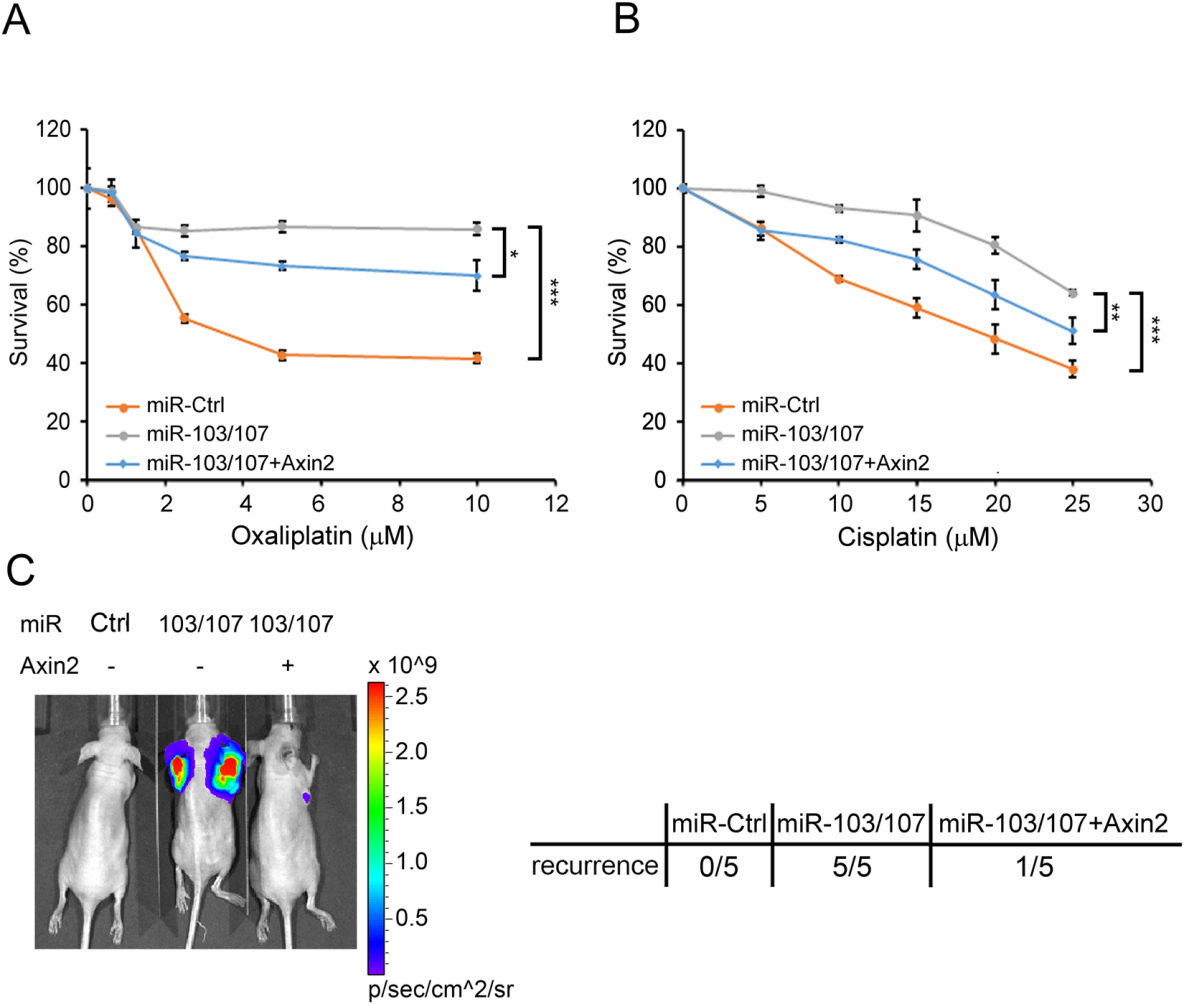
miR-103/107-Axin2 axis promotes chemoresistance and tumor recurrence. (**A**,**B**) Viability of indicated HCT116 derivatives in response to the treatment of oxaliplatin (**A**) or cisplatin (**B**). Data are mean ± S.D. *P < 0.05, **P < 0.005, ***P < 0.0005, n = 3. (**C**) Tumor-recurrence incidence was measured by subcutaneously implanting HCT116 derivatives carrying firefly luciferase into nude mice. The primary tumors were removed when they reached to 50 mm^3^. Eight weeks later, tumor recurrence at the primary site or distant regions was monitored by bioluminescent imaging using IVIS image system. Representative IVIS image is shown on the left panel, whereas the incidence of tumor recurrence is on the right panel.

CSCs play crucial roles in driving tumor recurrence and therapy resistance. We therefore tested the impact of miR-103/107-Axin2 axis on these characteristics. Compared with control cells, HCT116 cells overexpressing miR-103/107 were more resistant to oxaliplatin and cisplatin, two chemotherapeutic agents for treating CRC (Fig. 6A,B). These chemoresistant effects of miR-103/107 were partially inhibited by Axin2 reexpression. To evaluate tumor recurrence, we implanted miR-103/107 derivatives subcutaneously into nude mice. When tumors reached to 50 mm^3^, they were removed by surgical operation and tumor recurrence was monitored at 8 weeks later. While recurrence was not observed in mice injected with control cells, mice injected with miR-103/107-overexpressing cells all exhibited tumor recurrence (Fig. 6C). Reconstitution of Axin2 expression in miR-103/107-overexpressing cells blocked miR-103/107-induced tumor recurrence. These results demonstrated the roles of miR-103/107-Axin2 axis in potentiating CRC recurrence and chemoresistance.

### miR-103/107-Axin2 axis is manifested in CRC patients and correlates with poor survival

**Figure 7:**
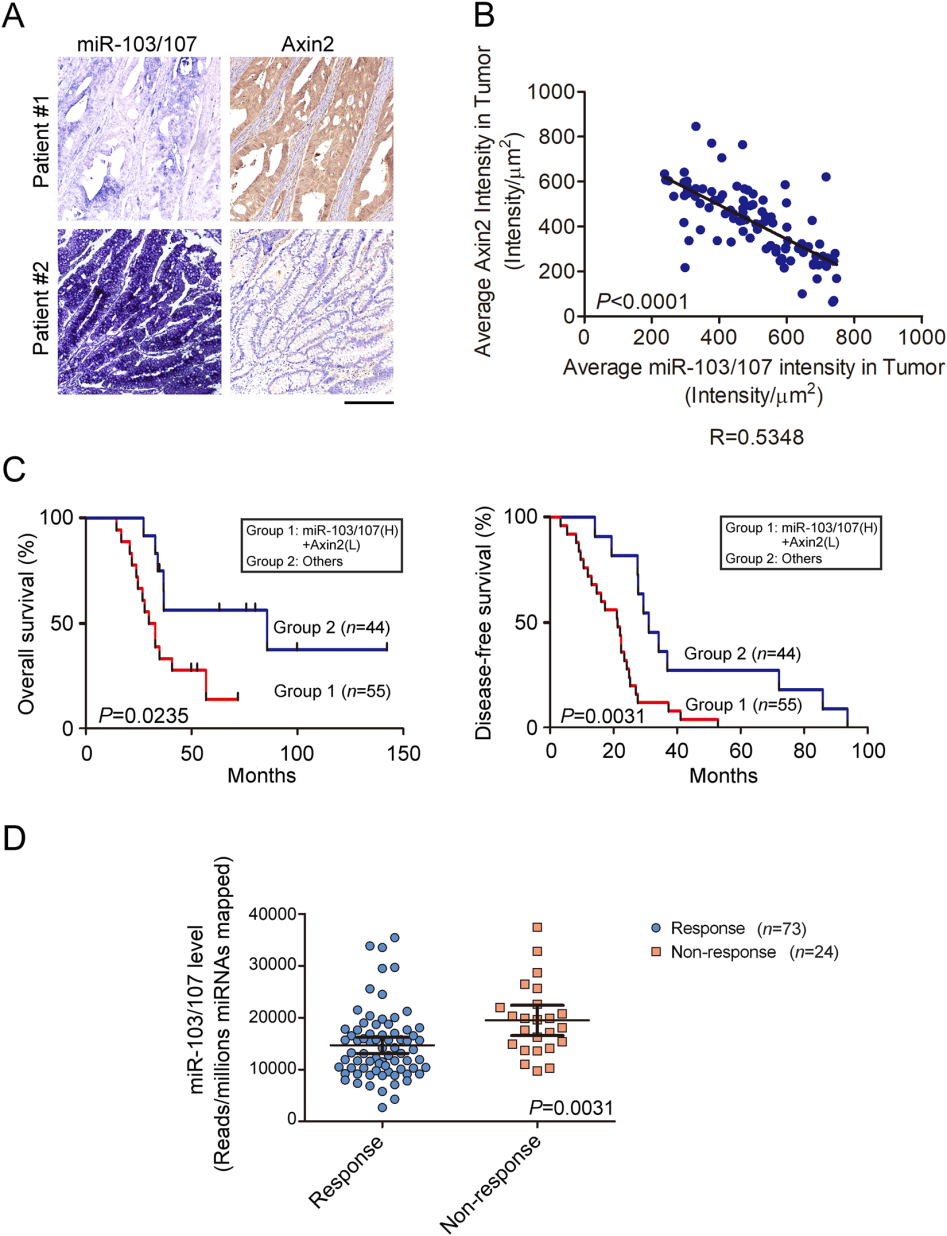
MiR-103/107-Axin2 axis correlates with poor prognosis of CRC patients. (**A**) Representing ISH staining for miR-103/107 expression and IHC staining for Axin2 expression in two CRC patient specimens. Bar, 50 mm. (**B**) Inverse correlation of miR-103/107 expression and Axin2 expression in CRC specimens. Pearson correlation test was used for comparison between groups. (**C**) Kaplan–Meier analysis of overall survival and disease-free survival of CRC patients with the corresponding expression profiles. Log-Rank test was used for comparison between groups. (**D**) Comparison of miR-103/107 level by response of patients to oxaliplatin treatment. Response group includes complete or partial response, while non-response one includes stable or progressive disease. Data are mean ± S.D.

To evaluate the clinical relevance of miR-103/107-induced Axin2 repression, we analyzed Axin2 and miR-103/107 expression by immunohistochemistry (IHC) and *in situ* hybridization (ISH), respectively, on consecutive slides derived from a cohort of CRC specimens. Representative images from CRC specimens were presented in Fig. 7A. This analysis revealed that miR-103/107 expression correlated inversely with Axin2 expression (Fig. 7B), suggesting the existence of miR-103/107-dependent downregulation of Axin2 in this cohort of CRC patients. More importantly, patients with a combined miR-103/107 high and Axin2 low expression signature had a poor overall survival and disease-free survival compared to other patients (Figs 7C and S4). Furthermore, consistent with a role in chemoresistant determination, miR-103/107 expression was higher in tumors from patients who didn’t respond to oxaliplatin versus who did (Fig. 7D). These data support a clinical relevance of miR-103/107-Axin2 axis in CRC and an association of its hyperactivation with poor prognosis.

## Discussion

In this study, we identify a new function of miR-103/107 in promoting multiple stem-like properties of CRC, including expression of stem-like markers, appearance of side-population cells, and capabilities in self-renewal, tumor initiation, tumor recurrence and chemoresistance. Mechanistically, these functions of miR-103/107 are mediated by upregulating Wnt/β-catenin signaling. We further identify Axin2 as a target of miR-103/107. By repressing Axin2, miR-103/107 facilitate a persistent induction of Wnt/β-catenin signaling and Wnt responsive genes. Since several Wnt responsive genes contribute to CRC stemness^36–38^, suppression of Axin2 is likely a prime mechanism for the stemness-promoting function of miR-103/107. In support of this notion, enforced Axin2 expression blocks the ability of miR-103/107 to enhance stem-like features in CRC. Our study thus identifies a critical role of miR-103/107-dependent repression of Axin2 in CRC stemness. Although miR-107-dependent repression of Axin2 was previously reported to facilitate cell proliferation in hepatocellular carcinoma^39^, our study further extends the impact of this pathway to CRC stemness. Given the tight association of stem-like properties of cancer cells with tumor initiation, progression, metastasis and therapy resistance, miR-103/107-Axin2 axis is likely a potential target for treating aggressive CRC.

We show that the expression of miR-103/107 correlates inversely with that of Axin2 in a cohort of CRC patients, demonstrating the clinical relevance of miR-103/107- Axin2 axis in CRC. This finding also identifies miRNA-mediated repression as a new mechanism for Axin2 downregulation in CRC, complementary to the previously reported promoter hypermethylation mechanism^16,17^. Consistent with the promoting role of miR-103/107- Axin2 axis in CRC stemness, we reveal a correlation of miR-103/107 high and Axin2 low expression profile with poor overall and disease-free survival, indicating the prognostic value of this axis.

While current study reveals the stemness-enhancing function of miR-103/107, our previous study identified the ability of these two miRNAs in promoting CRC migration, invasion, and metastasis by repressing DAPK and KLF4^23^. Thus, miR-103/107 would endow tumor cells with both stem-like features and invasive ability which could act in concert to facilitate the generation of circulating tumor cells (CTCs) with stem-like properties, a cell population with a high incidence of colonization at the distant sites. In line with this notion, miR-103 and/or miR-107 have been detected in the circulation of patients with CRC or other cancer types. More importantly, the presence of circulating miR-103/107 is associated with poor survival, recurrence, and lymph node metastasis in many cancer types^18–21,40^. Since such circulating miR-103/107 could be originated from CTCs carrying these miRNAs, the stemness-promoting function of miR-103/107 might contribute in part to the linkage between circulating miR-103/107 and poor prognosis.

Previous studies indicated that the expression levels of miR-103 and miR-107 are elevated under hypoxia^23,25^, a condition frequently occurring in the tumor microenvironment. Thus, the miR-103/107-induced Axin2 downregulation and sustained activation of Wnt/β-catenin signaling should be further aggravated in hypoxia conditions. In line with this notion, a previous report demonstrated the promoting effect of hypoxia on Wnt signaling and CRC stemness^41,42^. It would be important to substantiate the contribution of miR-103/107 to hypoxia-induced Wnt activation.

In summary, we show that miR-103/107 target Axin2 to lead to a sustained Wnt/β-catenin signaling, thereby promoting multiple stem-like features of CRC. Clinically, the miR-103/107-Axin2 axis is manifested in CRC patients and its hyperactivation correlates with poor prognosis. Our study thus supports a rational design of targeting miR-103/107-Axin2 axis for CRC treatment.

## Materials and Methods

### Cell culture and transfection

HCT116 and HT29 cells were obtained from American Type Culture Collection and cultured in RPMI-1640 medium containing 10% fetal calf serum (FCS). 293FT cells were maintained in Dulbecco’s Modified Eagle’s Medium (DMEM) supplemented with 10% FCS. Transfection was carried out by Lipofectamine 3000 Reagent (Invitrogen) or calcium phosphate method. Stable cell lines were generated by lentivirus transduction as described previously^23^.

### Plasmids

Plasmid encoding miR-103/107 sponge was constructed by placing 12 copies of miR-103/107 binding site downstream of a *GFP* gene in a lentivirus-based vector with a 6-nt spacer between each binding site. A 3-nt mismatch bulged region that prevents cleavage of the sponge transcript was placed in the center of each binding site as described previously^28^. To generate *Axin2* 3′-UTR construct, *Axin2* 3′-UTR fragment was amplified from the genomic DNA of 293 T cells and then inserted into psiCHECK-2 vector. *Axin2* 3′-UTR mutants were generated by site-directed mutagenesis. The Axin2 expression constructs were generated by inserting *Axin2* cDNA fragment amplified from HCT116 cells into pRK5 vector and lentivirus-based vector.

### Antibodies and reagents

Antibodies used in this study were obtained from commercial sources as follows: anti-β-catenin (610153; BD Transduction Laboratories), anti-Axin2 (76G2; Cell Signaling/WB; HPA042344; Sigma/IHC), anti-GFP (sc-9996; Santa Cruz), anti-GAPDH (Epitomics), anti-CD44-APC (C26; BD Pharmigen), APC mouse IgG2b k isotype control (BD Pharmigen), anti-DCLK1 (Abcam) and PE conjugated-goat anti-rabbit IgG H&L (Abcam). EGF, bFGF, and Wnt3a were obtained from R&D systems. Verapamil, Hoechst 33342, PI, KY02111, cisplatin, and oxaliplatin were purchased from Sigma. miR-103/107 precursors and miR-103/107 LNAs (antagomirs) were purchased from Ambion and EXIQON, respectively.

### RNAi interference

Knockdown of Axin2 was performed by transfection with siRNA oligonucleotides obtained from Dharmacon. The target sequences for these siRNAs are Axin 2 siRNA#1 5′-AAUGGUGGUGGUGGUGGUGUU-3′ and Axin2 siRNA#2 5′-AAAUGCGUGGAUACCUUAGAC-3′.

### Establishment of stable cell lines expressing miRNA sponges

Lentiviruses carrying miR-103/107 sponge and control sponge were generated as previously described^23^. After infection, cells were selected for the integration of sponge expression element by blasticidin (1 μg/ml). Notably, the miRNA sponge absorbs endogenous miRNA to inhibit the translation of GFP reporter gene containing multiple miRNA binding sites at its 3′-UTR. Only when the miRNA sponge is expressed at a level exceeding a threshold, the stable transfectants can become GFP+ cells. To enrich this population of cells, blasticidin-resistant cells were sorted with GFP high expression by FACS. The resulting population is considered as the miR-103/107 sponge expressing line.

### Sphere formation assay

To monitor sphere formation, HCT116 and HT29 stable clones were plated on 6-well ultra-low attachment plates at a density of 500 cells per well and cultured in tumor sphere medium as describe previously^43^. After 2~3 weeks, tumor spheres were imaged by an inverted phase-contrast microscope (Olympus) equipped with a CCD camera (Olympus). To quantify the sphere forming ability, cells were plated on 96-well ultra-low attachment plates at a density of 1 cell per well and maintained in tumor sphere medium. After 14 days, the percentage of wells containing tumor spheres was measured.

### Flow cytometry analysis

To assess the cell surface expression of stem cell markers CD44 and DCLK1, trypsinized cells using dissociation buffer (GIBCO) were blocked with Ca^2+^, Mg^2+^-free HBSS containing 2% goat serum. Cells were then incubated with primary antibody for 1 hr at 4 °C, followed by fluorophore-conjugated secondary antibody for at least 30 min at 4 °C. After labeling dead cells by propidium iodide staining, cells were washed and analyzed on a Becton Dickinson FACScan flow cytometer. To assay the side-populated cells with capability to efflux the DNA-binding dye Hoechst 33342, 1 × 10^6^ cells were incubated with pre-warmed growth medium containing 5 mg/ml Hoechst 33342 at 37 °C for 2 hrs. In the control experiments, cells were incubated in the presence of 100 mM verapamil to block Hoechst 33342 efflux. After incubation, cells were washed and subjected to flow cytometry analysis.

### Luciferase assay

For 3′-UTR analysis, HCT116 derivatives were transfected with psiCHECK-2-based construct containing *Axin2* 3′-UTR downstream of the *Renilla* luciferase gene. The luciferase activity was assayed with the Dual-Luciferase Reporter Assay System (Promega) at 48 hr post transfection.

### RT-qPCR

Total RNAs were extracted from cells by TRIZOL reagent (Invitrogen) and quantified by NanoDrop (Thermo SCIENTIFIC). Reverse transcription was performed using iScript™ cDNA Synthesis Kit (Bio-Rad) according to the manufacturer’s instructions.

Quantitative real-time PCR was performed on LightCycler® 480 System with SYBR Green I Master kit (Roche). To normalize the loading of cDNA, GAPDH was used as an internal control. The qPCR primers used in this analysis were ASCL2 forward: 5′-CGTGAAGCTGGTGAACTTGG-3′; ASCL2 reverse: 5′-GGATGTACTCCACGGCTGAG-3′; Myc forward: 5′-GGCTCCTGGCAAAAGGTCA-3′; Myc reverse: 5′-CTGCGTAGTTGTGCTGATGT-3′; S100A4 forward: 5′-GATGAGCAACTTGGACAGCAA-3′; S100A4 reverse: 5′-CTGGGCTGCTTATCTGGGAAG-3′; PLAUR forward: 5′-AAGCTATATGGTAAGAGGCTGTGC-3′; PLAUR reverse: 5′-CCACTTTTAGTACAGCAGGAGACA-3′; GAPDH forward: 5′-TGTTGCCATCAATGACCCCTT-3′; and GAPDH reverse: 5′-CTCCACGACGTACTCAGCG-3′.

### Cell viability (MTS) assay

Cells were seeded at a density of 3 × 10^3^ cells in 96-well plates and treated with cisplatin or oxaliplatin for 48 hr. Cell viability after drug treatment was assessed by adding Cell Titer 96 Aqueous One Solution Reagent (Promega), followed by measuring the Absorbance_490 nm_ using Paradigm ELISA reader.

### Xenograft models and tumor recurrence

All mouse experiments in this study were conducted with approval from the Experimental Animal Committee, Academia Sinica. Six-week-old female BALB/c nude mice were purchased from National Laboratory Animal Center (Taipei, Taiwan) and were maintained under specific pathogen-free conditions. For tumor-initiation ability, HCT116 and HT29 derivatives mixed with Matrigel were injected subcutaneously at various cell numbers. Tumor incidence and tumor initiation ability were monitored at seven weeks after injection. For tumor recurrence, HCT116 derivatives carrying firefly luciferase were injected subcutaneously into nude mice to induce primary tumors. After tumors’ size reaching ~50 mm^3^, the tumors were removed by surgery and the recurrence was monitored by bioluminescent imaging using IVIS image system. All animal procedures were conducted according to the guidelines of the Institutional Animal Care and Use Committee (IACUC).

### ISH and IHC staining

99 CRC specimens with survival and recurrence information were obtained from US Biomax Inc. and Taipei Medical University Tissue Bank. Studies involving these tissues were approved by the Institutional Review Boards at Taipei Medical University and Academia Sinica. The informed consent of these tissues were specified waived by institutional review board. *In situ* hybridization analysis with 3′ DIG-labeled miRNA-103 LNA and miR-107 LNA (mixed at 1:1 ratio) and immunohistochemical analysis with avidin-biotin-peroxidase method were described previously^23^. Slide images were acquired by scanner microscope at 20X microscopic magnification (3DHISTECH). Staining intensity of Axin2 and miR-103/107 were analyzed by HistoQuest3.5 software (TissueGnostics) using Single Reference Shade module for color separation and Total Area Measurement option for quantification as described previously^44^.

### Analysis of miR-103/107 level from miRNA-seq database

To evaluate miR-103/107 association to chemoresistance in CRC patients, miRNA-seq expression profile and detailed clinical information from CRC patientes were downloaded from The Cancer Genome Atalas (https://cancergenome.nih.gov/). The miR-103/107 level of tumor from patients who respond to oxaliplatin or not were retrieved from the reads of per million miRNAs mapped. Patients’ response to oxaliplatin were categorized into two groups: response and non-response that include complete or partial response and stable or progressive disease, respectively.

## Supplementary information

Supplement Information

## References

[1] Ferlay J (2015). Cancer incidence and mortality worldwide: Sources, methods and major patterns in GLOBOCAN 2012. International Journal of Cancer.

[2] Clarke MF (2006). Cancer stem cells–perspectives on current status and future directions: AACR Workshop on cancer stem cells. Cancer Res.

[3] Vermeulen L, Sprick MR, Kemper K, Stassi G, Medema JP (2008). Cancer stem cells–old concepts, new insights. Cell Death Differ.

[4] Kim Y, Lin Q, Glazer PM, Yun Z (2009). Hypoxic Tumor Microenvironment and Cancer Cell Differentiation. Current molecular medicine.

[5] Mani SA (2008). The Epithelial-Mesenchymal Transition Generates Cells with Properties of Stem Cells. Cell.

[6] Gregorieff A, Clevers H (2005). Wnt signaling in the intestinal epithelium: from endoderm to cancer. Genes & Development.

[7] Korinek V (1998). Depletion of epithelial stem-cell compartments in the small intestine of mice lacking Tcf-4. Nat Genet.

[8] Liu C (1999). beta-Trcp couples beta-catenin phosphorylation-degradation and regulates Xenopus axis formation. Proc Natl Acad Sci USA.

[9] Daniels DL, Weis WI (2005). Beta-catenin directly displaces Groucho/TLE repressors from Tcf/Lef in Wnt-mediated transcription activation. Nat Struct Mol Biol.

[10] He TC (1998). Identification of c-MYC as a target of the APC pathway. Science.

[11] Jubb AM (2006). Achaete-scute like 2 (ascl2) is a target of Wnt signalling and is upregulated in intestinal neoplasia. Oncogene.

[12] Lustig B (2002). Negative feedback loop of Wnt signaling through upregulation of conductin/axin2 in colorectal and liver tumors. Mol Cell Biol.

[13] Herbst A (2014). Comprehensive analysis of β-catenin target genes in colorectal carcinoma cell lines with deregulated Wnt/β-catenin signaling. BMC Genomics.

[14] Bernkopf DB, Hadjihannas MV, Behrens J (2015). Negative-feedback regulation of the Wnt pathway by conductin/axin2 involves insensitivity to upstream signalling. Journal of Cell Science.

[15] Chen Baozhi, Dodge Michael E, Tang Wei, Lu Jianming, Ma Zhiqiang, Fan Chih-Wei, Wei Shuguang, Hao Wayne, Kilgore Jessica, Williams Noelle S, Roth Michael G, Amatruda James F, Chen Chuo, Lum Lawrence (2009). Small molecule–mediated disruption of Wnt-dependent signaling in tissue regeneration and cancer. Nature Chemical Biology.

[16] Koinuma K (2006). Epigenetic silencing of AXIN2 in colorectal carcinoma with microsatellite instability. Oncogene.

[17] Muto Y (2014). DNA methylation alterations of AXIN2 in serrated adenomas and colon carcinomas with microsatellite instability. BMC Cancer.

[18] Kleivi Sahlberg K (2015). A serum microRNA signature predicts tumor relapse and survival in triple-negative breast cancer patients. Clin Cancer Res.

[19] Nonaka R (2015). Circulating miR-103 and miR-720 as novel serum biomarkers for patients with colorectal cancer. Int J Oncol.

[20] Stuckrath I (2015). Aberrant plasma levels of circulating miR-16, miR-107, miR-130a and miR-146a are associated with lymph node metastasis and receptor status of breast cancer patients. Oncotarget.

[21] Treece AL (2016). Gastric adenocarcinoma microRNA profiles in fixed tissue and in plasma reveal cancer-associated and Epstein-Barr virus-related expression patterns. Lab Invest.

[22] Zhao JY (2015). Five miRNAs Considered as Molecular Targets for Predicting Esophageal Cancer. Med Sci Monit.

[23] Chen H-Y (2012). miR-103/107 Promote Metastasis of Colorectal Cancer by Targeting the Metastasis Suppressors DAPK and KLF4. Cancer Research.

[24] Zheng YB (2016). MicroRNA-103 promotes tumor growth and metastasis in colorectal cancer by directly targeting LATS2. Oncol Lett.

[25] Chen Z (2013). Hypoxia-responsive miRNAs target argonaute 1 to promote angiogenesis. The Journal of Clinical Investigation.

[26] Martello G (2010). A MicroRNA targeting dicer for metastasis control. Cell.

[27] Chen P-S (2011). miR-107 promotes tumor progression by targeting the let-7 microRNA in mice and humans. The Journal of Clinical Investigation.

[28] Ebert MS, Neilson JR, Sharp PA (2007). MicroRNA sponges: competitive inhibitors of small RNAs in mammalian cells. Nat Methods.

[29] Farrall AL (2012). Wnt and BMP signals control intestinal adenoma cell fates. Int J Cancer.

[30] Sadanandam A (2013). A colorectal cancer classification system that associates cellular phenotype and responses to therapy. Nat Med.

[31] Minami I (2012). A Small Molecule that Promotes Cardiac Differentiation of Human Pluripotent Stem Cells under Defined, Cytokine- and Xeno-free Conditions. Cell Reports.

[32] John B (2004). Human MicroRNA targets. PLoS Biol.

[33] Grimson A (2007). MicroRNA targeting specificity in mammals: determinants beyond seed pairing. Mol Cell.

[34] Krek A (2005). Combinatorial microRNA target predictions. Nat Genet.

[35] Jho EH (2002). Wnt/beta-catenin/Tcf signaling induces the transcription of Axin2, a negative regulator of the signaling pathway. Mol Cell Biol.

[36] Wong DJ (2008). Module Map of Stem Cell Genes Guides Creation of Epithelial Cancer Stem Cells. Cell Stem Cell.

[37] Barker N (2007). Identification of stem cells in small intestine and colon by marker gene Lgr5. Nature.

[38] Zhu R (2012). Ascl2 Knockdown Results in Tumor Growth Arrest by miRNA-302b-Related Inhibition of Colon Cancer Progenitor Cells. PLoS ONE.

[39] Zhang JJ (2015). miR-107 promotes hepatocellular carcinoma cell proliferation by targeting Axin2. Int J Clin Exp Pathol.

[40] Zhao Q (2015). Circulating miRNAs is a potential marker for gefitinib sensitivity and correlation with EGFR mutational status in human lung cancers. Am J Cancer Res.

[41] Dong HJ (2016). The Wnt/beta-catenin signaling/Id2 cascade mediates the effects of hypoxia on the hierarchy of colorectal-cancer stem cells. Sci Rep.

[42] Liu HL, Liu D, Ding GR, Liao PF, Zhang JW (2015). Hypoxia-inducible factor-1alpha and Wnt/beta-catenin signaling pathways promote the invasion of hypoxic gastric cancer cells. Mol Med Rep.

[43] Johnson, S., Chen, H. & Lo, P. K. *In vitro* Tumorsphere Formation Assays. *Bio Protoc* **3** (2013). 10.21769/bioprotoc.325 PMC4972326 27500184

[44] Puhr M (2009). Down-regulation of suppressor of cytokine signaling-3 causes prostate cancer cell death through activation of the extrinsic and intrinsic apoptosis pathways. Cancer Res.

