# Supplementary material for: miR-103/107 prolong Wnt/β-catenin signaling and colorectal cancer stemness by targeting Axin2
Source: Sci Rep. 2019 Jul 4;9:9687. doi: 10.1038/s41598-019-41053-z (PMC6609830; doi:10.1038/s41598-019-41053-z)
Supplement: Supplementary file 1 — Supplement Information [file 41598_2019_41053_MOESM1_ESM.pdf]

Supplemental information for

miR-103/107 prolong Wnt/β-catenin signaling and colorectal cancer stemness by targeting Axin2

Hsin-Yi Chen[1, 2, 7, 8], Yaw-Dong Lang[4], Han-Nan Lin[1], Yun-Ru Liu[6], Chun-Chieh Liao[2,3], André Wendindondé Nana[1], Yun Yen[1, 8], and Ruey-Hwa Chen[2, 3, 5]

[1]Ph.D Program for Cancer Molecular Biology and Drug Discovery, College of Medical Science and Technology, Taipei Medical University, [2]Institute of Biological Chemistry, Academia Sinica, [3]Institute of Molecular Medicine, College of Medicine, National Taiwan University, [4]Institute of Biomedical Science, Academia Sinica, [5]Institute of Biochemical Sciences, College of Life Science, National Taiwan University, Taipei, Taiwan. [6]Office of Human Research, Taipei Medical University, Taipei, Taiwan. [7]Graduate Institute of Cancer Biology and Drug Discovery, College of Medical Science and Technology, Taipei Medical University, [8]TMU Research Center of Cancer Translational Medicine.

Correspondence to: Hsin-Yi Chen, email: hyichen@tmu.edu.tw ; Ruey-Hwa Chen, email: rhchen@gate.sinica.edu.tw

Supplemental Data

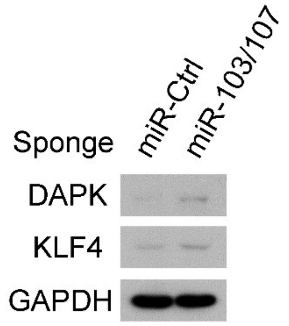

Figure S1. The efficacy of miR-103/107 sponge. Immunoblot analysis of DAPK and KLF4 in HCT116 cells stably expressing indicated miRNA sponges. GAPDH was used as a loading control.

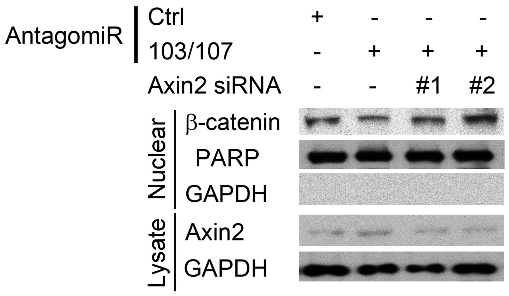

Figure S2. miR-103/107 induce β-catenin nuclear accumulation via regulating Axin2 mRNA. Immunoblot analysis of Axin2 and nuclear β-catenin levels in HT29 cells transiently transfected with indicated antagomiRs and/or siRNA.

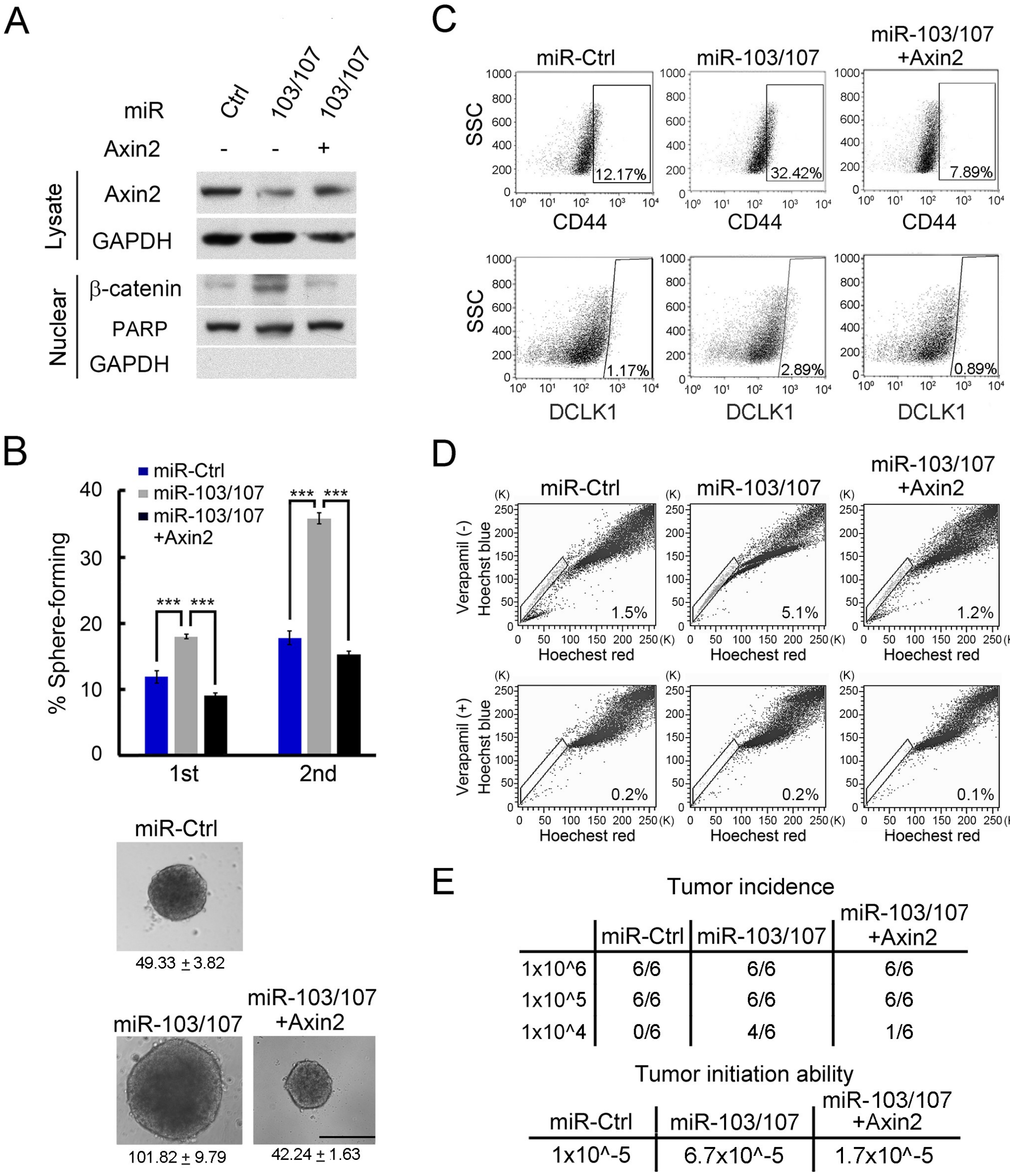

Figure S3. Suppression of Axin2 by miR-103/107 promotes CRC stemness. A. Axin2 and nuclear β-catenin levels in HT29 cells expressing indicated miRNAs and Axin2 construct were assayed by immunoblot analysis. **B**. Tumor sphere-forming abilities of HT29 derivatives as shown in **(A)**. Representative images of spheres and sphere sizes (calculated from 30 spheres per group experiment) are shown on the right

panel. The images were taken at 3 weeks after cell plating. Quantitative data of sphere number are shown on the left panel. Scale bar, 50 mm. Data are mean ± S.D. ***: $P < 0.0005$, n = 3. **C-D.** HT29 derivative as shown in **(A)** were assayed by flow cytometry for cell surface expression of stem-like markers CD44 (upper panel) and DCLK1 (lower panel) **(C)**, and side-population cells **(D)**. **E.** Tumor-initiation ability was measured by subcutaneously implanting HT29 derivatives as shown in **(A)** at indicated cell numbers into nude mice. Seven weeks after transplantation, tumor incidence and tumor-initiating ability were measured.

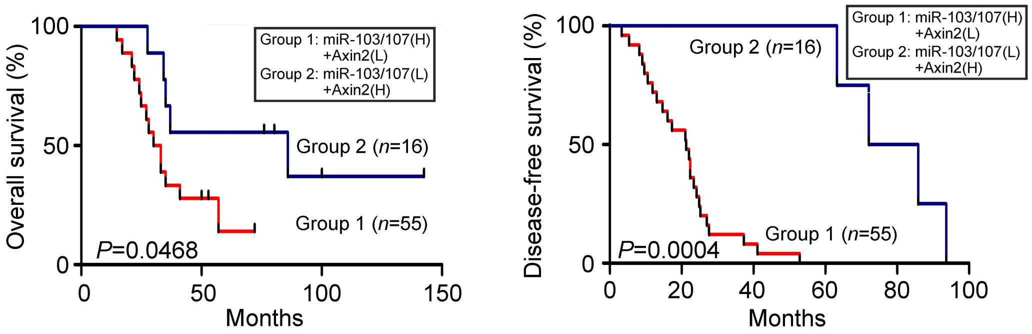

Figure S4. miR-103/107-Axin2 axis correlates with poor prognosis of CRC patients. Kaplan–Meier analysis of overall survival and disease-free survival of CRC patients with the corresponding expression profiles. Log-Rank test was used for comparison between groups.

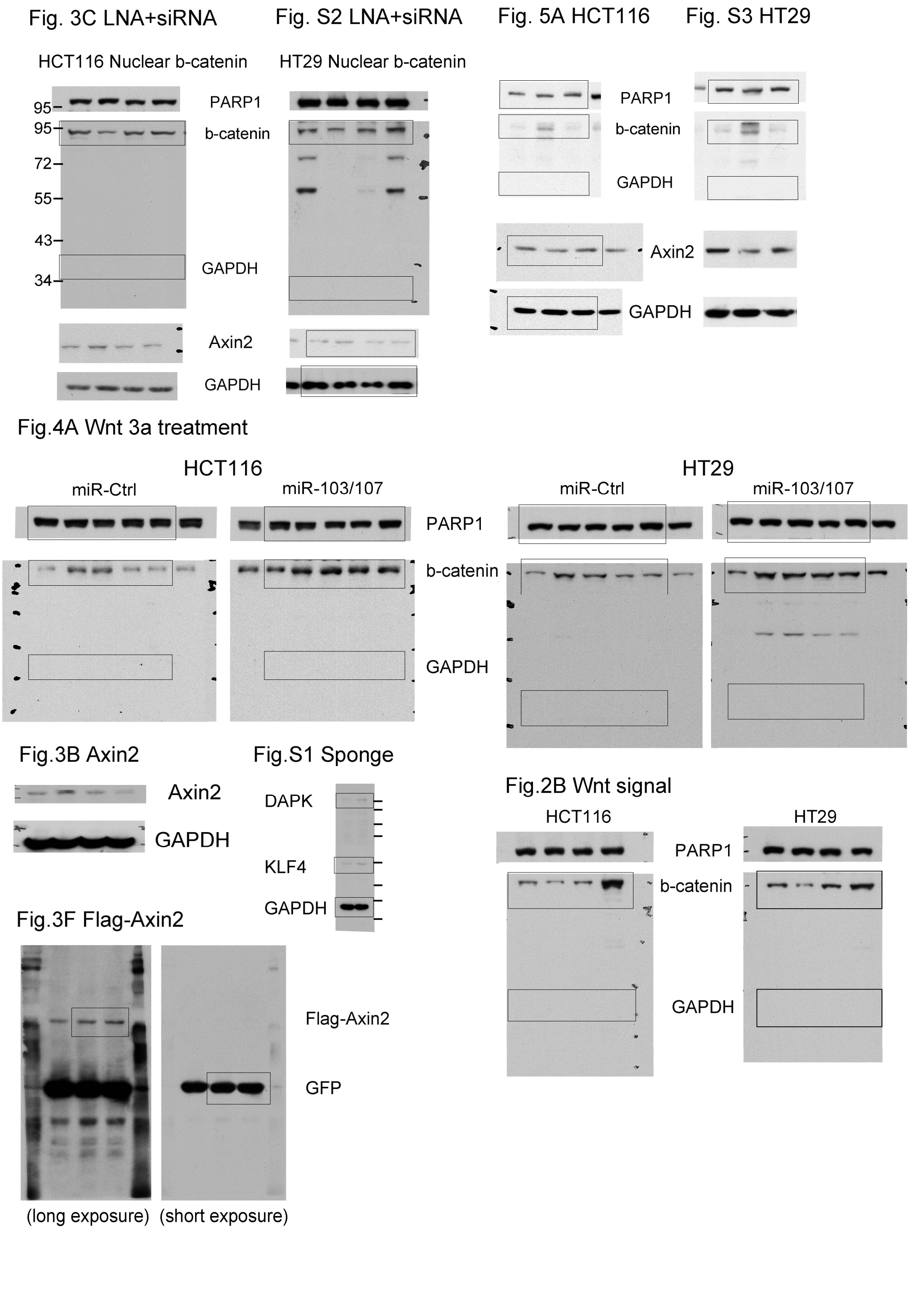

Figure S5. Full scans of western blot data shown in Fig. 2-5, S1-S3.
